# Integrative Analysis of Triphala’s Therapeutic Mechanisms in Periodontitis: Network Pharmacology-guided Investigation with Experimental Validation in Cellular and Animal Models

**DOI:** 10.3290/j.ohpd.c_2572

**Published:** 2026-05-28

**Authors:** Yiwei Zhao, Simin Li, Deborah Kreher, Rainer Haak, Andreas Fichter, Gerhard Schmalz

**Affiliations:** a Yiwei Zhao Dentist, Department of Craniomaxillofacial Surgery, Leipzig University, Leipzig 04109, Germany. Research design, conducted the network pharmacological analyses, performed the experiments, analysed the data, drafted the manuscript, read and approved the final manuscript.; b Simin Li Dentist, Stomatological Hospital, School of Stomatology, Southern Medical University, 366 Jiangnan South Avenue, Haizhu district, Guangzhou 510280, Guangdong Province, China. Research design, conducted the network pharmacological analyses, performed the experiments, analysed the data, drafted the manuscript, read and approved the final manuscript.; c Deborah Kreher Dentist, Department of Conservative Dentistry and Periodontology, Brandenburg Medical School (MHB) Theodor Fontane, Brandenburg an der Havel, Germany. Analysed the data, read and approved the final manuscript.; d Rainer Haak Dentist, Department of Cariology, Endodontology and Periodontology, Leipzig University, Leipzig 04109, Germany. Analysed the data, read and approved the final manuscript.; e Andreas Fichter Dentist, Department of Craniomaxillofacial Surgery, Leipzig University, Leipzig 04109, Germany. Administered and supervised the whole research project, read and approved the final manuscript.; f Gerhard Schmalz Dentist, Department of Conservative Dentistry and Periodontology, Brandenburg Medical School (MHB) Theodor Fontane, Brandenburg an der Havel, Germany. Research design, administered and supervised the whole research project, read and approved the final manuscript. Yiwei Zhao and Simin Li contributed equally as the first author. Andreas Fichter and Gerhard Schmalz contributed equally as the corresponding author and senior author.

**Keywords:** anti-inflammatory, antioxidative stress, fibroblasts, network pharmacology, periodontitis, periodontal ligament, PI3K/AKT signalling pathway, RANKL/OPG, Triphala

## Abstract

**Purpose:**

Periodontitis is a chronic inflammatory disease characterised by progressive destruction of periodontal tissues and alveolar bone resorption. Triphala (TRP), a traditional Ayurvedic formulation comprising equal proportions of *Terminalia chebula*, *Terminalia bellirica*, and *Phyllanthus emblica*, has demonstrated anti-inflammatory and antioxidant properties. This study aimed to investigate the molecular mechanisms underlying TRP’s therapeutic effects on periodontitis through an integrated approach combining network pharmacology with experimental validation, focusing on the *PI3K*/*AKT* signalling pathway.

**Methods and Materials:**

Network pharmacology analysis was performed using TCMSP and TCM databases to identify active compounds and potential targets of TRP. The intersection between TRP targets and periodontitis-related genes was analysed. *In vitro* studies utilised lipopolysaccharide (LPS)-induced human periodontal ligament fibroblasts (hPDLFs) treated with various concentrations of TRP (5–40 μg/ml). Cell viability (CCK-8), reactive oxygen species (ROS) levels (flow cytometry), and expression of hub genes, oxidative stress markers, and *PI3K*/AKT pathway components were assessed via Western blotting and qPCR. Functional rescue experiments using *PI3K* activator (740Y-P) and inhibitor (LY294002) were conducted. *In vivo* validation employed a ligature-induced periodontitis rat model with TRP irrigation treatment, evaluated through micro-CT, histological staining (H&E and TRAP), and molecular analyses.

**Results:**

Network pharmacology identified 129 potential targets of TRP for treating periodontitis, with *PI3K*/*AKT* emerging as a key signalling pathway. The top 10 hub genes included *JUN*, *TP53*, *MYC*, *EGFR*, and *AKT1*. TRP (20 μg/ml) significantly restored LPS-induced cell viability reduction (P < 0.01) and decreased ROS levels (P < 0.01). TRP downregulated the expression of hub genes (P53, *MYC*, *EGFR*, *AKT1*) and oxidative stress markers (SOD, CAT, Nrf2, HO-1) elevated by LPS (P < 0.01). Mechanistically, TRP suppressed *PI3K*/*AKT* pathway activation, reducing phosphorylated *PI3K*, *AKT1*, and *AKT2* levels while upregulating *PTEN* expression (P < 0.01). These effects were reversed by the *PI3K* activator and enhanced by the *PI3K* inhibitor. TRP treatment significantly decreased inflammatory cytokines (IL-1β, IL-6, TNF-α) and MMP8 secretion (P < 0.01). *In vivo*, TRP irrigation reduced alveolar bone loss (decreased ABC-CEJ distance, increased BV/TV ratio, P < 0.05), decreased RANKL/OPG ratio (2.3 ± 0.242 vs 8.481 ± 1.56 in model group, P < 0.05), reduced osteoclast numbers (P < 0.05), and attenuated inflammatory cell infiltration in periodontal tissues.

**Conclusions:**

This study demonstrates that TRP exerts anti-inflammatory and anti-oxidative effects on LPS-induced periodontal inflammation through inhibition of the *PI3K*/*AKT* signalling pathway. The integration of network pharmacology with comprehensive experimental validation reveals TRP’s multi-target therapeutic mechanisms in periodontitis. These findings provide scientific evidence supporting TRP as a promising natural therapeutic agent for periodontal disease management and suggest its potential for development as an adjunctive treatment in clinical periodontal therapy.

Periodontitis is one of the most prevalent chronic inflammatory diseases worldwide, affecting approximately 50% of adults and representing the leading cause of tooth loss in adults over 40 years of age 14. The disease is characterised by progressive destruction of tooth-supporting structures, including the periodontal ligament, cementum, and alveolar bone, primarily initiated by dysbiotic microbial communities in subgingival biofilms.^20^ Lipopolysaccharide (LPS) from gram-negative bacteria, particularly *Porphyromonas gingivalis*, serves as a key pathogen-associated molecular pattern that triggers host inflammatory responses through Toll-like receptor 4 activation.^9^ This inflammatory cascade leads to increased production of pro-inflammatory cytokines, including interleukin-1β (IL-1β), tumour necrosis factor-α (TNF-α), and interleukin-6 (IL-6), which orchestrate the recruitment of immune cells and activation of osteoclastogenesis.^2^ Additionally, oxidative stress plays a crucial role in periodontitis pathogenesis, with reactive oxygen species (ROS) causing direct tissue damage and amplifying inflammatory responses.^25^ The RANKL/OPG system represents a critical regulatory axis in alveolar bone metabolism, with increased RANKL/OPG ratios correlating with enhanced osteoclastic activity and bone resorption in periodontitis.^26^ Current treatment modalities, including mechanical debridement and antimicrobial therapy, often fail to completely resolve inflammation or regenerate lost periodontal tissues, highlighting the need for novel therapeutic approaches.^1,14,15,21^

Recent systematic reviews have demonstrated that herbal medicines with anti-inflammatory, antimicrobial, and antioxidant properties, show considerable efficacy in improving oral health and treating gingivitis and periodontitis.^6,16^ Among these herbal formulations, Triphala (TRP), a traditional Ayurvedic polyherbal formulation consisting of equal proportions of three medicinal plants – *Terminalia chebula*, *Terminalia bellirica*, and *Phyllanthus emblica* – has demonstrated promising anti-inflammatory and antimicrobial properties in preliminary periodontal studies.^11,22^ Clinical trials have shown that TRP mouthwash can reduce plaque accumulation and gingival inflammation comparable to chlorhexidine, yet without the associated side effects such as tooth staining and taste alteration.^3^ Despite these clinical findings, the precise molecular targets and signalling pathways mediating TRP’s periodontal protective effects remain largely unexplored. Previous studies on individual components of TRP have suggested potential modulation of oxidative stress pathways and inflammatory signalling, but a comprehensive investigation of TRP’s multi-target mechanisms in periodontitis is lacking.^4^ Furthermore, the integration of network pharmacology approaches with experimental validation offers unprecedented opportunities to systematically elucidate the complex interactions between herbal compounds and disease-related molecular networks, yet this approach has not been applied to investigate TRP’s effects on periodontitis.^12^

Therefore, the present study aimed to comprehensively investigate the anti-inflammatory and antioxidative mechanisms of TRP in periodontitis through an integrated approach combining network pharmacology prediction with experimental validation. We employed systematic network analysis to identify potential targets and pathways, followed by *in vitro* experiments using LPS-induced human periodontal ligament fibroblasts and *in vivo* validation in a rat periodontitis model. This multi-level investigation focused particularly on elucidating TRP’s regulatory effects on the *PI3K*/*AKT* signalling pathway and its downstream inflammatory and oxidative stress responses.

## METHODS AND MATERIALS

### Identification of TRP’s Therapeutic Targets for Periodontitis Treatment

The main components of TRP are *Terminalia chebula* (Hezi), *Terminalia bellirica* (Maohezi), and *Phyllanthus emblica* / *Emblica officinalis* (Yuganzi).The small molecules of TRP components were obtained from two databases: Traditional Chinese Medicine Systems Pharmacology Database (TCMSP) and TCM Database@Taiwan (http://tcm.cmu.edu.tw/). The drug targets of TRP’s small molecules were predicted using TCMSP and TCM databases. For TCMSP, the prediction of the main small molecule drug targets of traditional Chinese medicine was based on two measures: oral bioavailability (OB) and drug-likeness (DL). For the prediction model, targets with OB ≥ 30% and DL ≥ 0.18 were selected as the main component drug targets of TRP. The downloaded targets were protein names, which were converted to gene names based on the Uniprot database (http://www.uniprot.org/). Periodontitis-related genes were obtained from four databases: DisGeNET (https://www.disgenet.org/home/), GeneCards (https://www.genecards.org/), OMIM (https://www.omim.org/), and PharmGkb (https://www.pharmgkb.org/). For GeneCards, genes with a relevance score ≥ 1 were selected as periodontitis-related genes. The union of genes obtained from these four data sets was considered as the predicted periodontitis-related genes. The intersection of the obtained periodontitis-related genes and TRP’s drug targets was taken, and the intersecting genes were considered as TRP’s drug targets for treating periodontitis.

### Functional Enrichment Analysis of Potential Drug Targets

This study performed functional enrichment analysis on the intersecting gene list of TRP drug targets and periodontitis-related genes found in the Venn diagram analysis. The analysis was conducted using R (version 4.2.1) and the clusterProfiler package (version 4.4.4). First, the intersecting gene list was converted to human (Homo sapiens) IDs using the org.Hs.eg.db conversion library. Then, based on the converted IDs and the clusterProfiler package, gene ontology enrichment analysis was performed on the intersecting gene list. Building upon the enrichment analysis results, further GOKEGG-clustering tree analysis was conducted to present the enrichment results more intuitively. The GOKEGG-clustering tree analysis was performed using R (version 4.2.1) and the ggplot2 package (version 3.3.6). The specific steps were as follows: first, the Jaccard similarity index between each pair of enrichment terms was calculated to obtain a similarity matrix; then, the hierarchical clustering algorithm hclust was used to perform clustering analysis on the matrix, yielding a clustering tree; finally, the ggplot2 package was employed to visualise the clustering tree, and the top 25 enrichment term IDs of interest were selected and displayed in the figure, ie, the top 25 enrichment term IDs with a corrected p-value less than 0.05 and sorted in ascending order.

### Construction of the TRP Drug Target Network for Periodontitis Treatment

The targets of TRP in treating periodontitis and their corresponding small molecules were extracted, and Cytoscape software was employed to construct the TRP drug target network. This network includes the TRP compound formula itself, the three herbal components that constitute TRP: *Terminalia chebula* (Hezi), *Terminalia bellirica* (Maohezi), and *Emblica officinalis* (Yuganzi), as well as the main active small molecule compounds contained in each flavouring herb. The network also encompasses the periodontitis-related genes targeted by these small molecule compounds. Finally, Cytoscape software was used to visualise the relationships among the above-mentioned components, small molecules, and targets, constructing a complete TRP drug target network.

### Construction and Analysis of the Protein–Protein Interaction (PPI) Network

Protein–protein interaction data was collected from multiple public databases, including BIOGRID (Biological General Repository for Interaction Datasets), HPRD (Human Protein Reference Database), DIP (Database of Interacting Proteins), MINT (Molecular INTeraction database), PINA (Protein Interaction Network Analysis), InnateDB (a knowledge resource for innate immunity interactions and pathways) and Instruct (3D protein interactome networks with structural resolution). Based on the potential target proteins of TRP in treating periodontitis obtained from small molecule target prediction and comparison with periodontitis-related genes, these target proteins and their interactions with other proteins were extracted from these databases. Next, using the Cytoscape biological network visualisation and analysis software, a protein–protein interaction network (PPI network) for TRP in treating periodontitis was constructed, where each node represents a protein molecule, and the edges represent direct physical interactions or functional regulatory relationships between two protein molecules. To deeply analyse the topological structural features of this PPI network, the NetworkAnalyzer plugin of Cytoscape was utilised to calculate and analyse key network topological parameters, including AverageShortestPathLength, BetweennessCentrality, ClosenessCentrality, ClusteringCoefficient, Degree, and Radiality. Through these parameters, the key proteins involved in the treatment process of TRP for periodontitis and their functional locations within the entire protein interaction network can be comprehensively evaluated.

### Functional Analysis of Drug Targets

To deeply explore the molecular mechanisms of TRP in treating periodontitis, the top 10 nodes with the highest degree values were extracted from the protein–protein interaction (PPI) network of TRP, representing the most critical target proteins. Then, using the Cytoscape plugins ClueGO and CluePedia, GO biological process and KEGG pathway enrichment analyses were performed on these target proteins, and enrichment terms with statistical significance (P.adjust ≤ 0.05) were selected. In the ClueGO analysis, since GO itself is a directed acyclic tree structure, GO terms can be divided into multiple levels. For pathway data without a hierarchical structure (such as KEGG and BioCarta), the level is set to –1; while GO levels 1–3 are generally common, containing a large number of genes and providing general biological information, levels 9–14 are more specific terms, with fewer related genes but more information and higher research value. ClueGO uses the kappa coefficient to measure the association strength between GO terms, grouping terms with high overlapping genes, and the higher the kappa value, the stronger the association. Finally, Cytoscape was used to plot the functional network diagram of the enriched GO terms and KEGG pathways for the target proteins. Meanwhile, the clusterProfiler package in R was also utilised to perform KEGG pathway enrichment analysis on the target proteins (P ≤ 0.05), and the results were visualised in the form of a bubble plot.

### Method for Preparing TRP Extracts

An extract of finely powdered TRP (Dabur India, Alwar, India; batch number: AL1675) containing equal amounts of *Terminalia chebula*, *Emblica officinalis*, and *Terminalia bellirica* (1:1:1) was prepared by refluxing ultrapure water (substrate/extractant: 1/10 (w/v), reflux, one h). After centrifugation (2860 g, 15 min), the precipitate was filtered through a 0.45-μm membrane filter (Merck Millipore, Cork, Ireland). A rotary evaporator and a lyophiliser were used to remove the filtrate solvent. An extract powder with a yellowish-green colour was weighed and kept at –20°C.

### In Vitro Cellular Experiments

#### Cell culture and treatment

Human periodontal ligament fibroblasts (hPDLFs) were purchased from Shanghai AnWei Biotechnology Co., (catalogue number AW-CH0506, Shanghai, China). The cells were cultured in DMEM medium supplemented with 10% foetal bovine serum and 1% penicillin-streptomycin. The cells were maintained in an incubator at 37°C with 5% CO_2_. hPDLF cells were subcultured two to three times per week.

#### Cell viability assay

The cultured cells were seeded into 96-well plates. They were then treated with 1 μg/ml LPS and TRP at concentrations of 5 μg/ml, 10 μg/ml, 20 μg/ml, and 40 μg/ml for 24 h. Control group cells were not treated with LPS. The culture plates were incubated at 37°C in a 5% CO_2_ incubator for 24 h. After incubation, the old culture medium was aspirated, and 100 μL of 10% Cell Counting Kit-8 (CCK-8) solution (Dojindo, CK04, Japan) was added to each well. The plates were then incubated in the incubator for 2 h. The absorbance at 450 nm was measured using a microplate reader.

#### Polymerase chain reaction (PCR) assay

To the aforementioned samples, 1 mL of TRIzol reagent was added to facilitate complete cell lysis. Subsequently, chloroform was introduced, and the mixture was thoroughly homogenised by vigorous shaking. Following centrifugation, the aqueous phase containing RNA was carefully extracted. The isolated RNA was then subjected to reverse transcription. The target genes for primers and AceQ qPCR SYBR Green Master Mix (YEASEN, China, 11202ES08) are *P53-140bp*, *MYC-159bp*, *EGFR-140bp*, *PTEN-153bp*, *PI3K-163bp*, *AKT1-150bp*, *AKT2-246bp*, *mTOR-295bp*, and *GSK-3*β*-122bp*. Amplification is performed in duplicate using Brilliant QPCR Master Mix (TransGen Biotech, China, AE301-03) and optimised primer/probe set concentrations in a 25 μl reaction system. The reaction conditions used in the ABI PRISM® 7500 Sequence Detection System (Applied Biosystems, America, ABI-7500 ) real-time fluorescence quantitative PCR instrument are as follows: 95°C for 5 min; 95°C for 15 s, 60°C for 32 s with plate reading, 40 cycles; melting curve analysis: temperature 60–95°C. The primers used are shown in Table 1.

**Table 1:** The primer sequence of all examined genes during the *in vitro* assays

Gene	Forward primer sequence	Reverse primer sequence
*PTEN*	5’-GTCAGAGGCGCTATGTGTATTA-3’	5’-TTAGCTGGCAGACCACAA-3’
*PI3K*	5’-AAAGGCGGCTTGAAAGGT-3’	5’-GACGATCTCCAATTCCCAAA-3’
*AKT1*	5’-ATCGCTTCTTTGCCGGTATC-3’	5’-CTTGGTCAGGTGGTGTGATG-3’
*AKT2*	5’-CGCAAGGTGTTAGCACTTCA-3’	5’-GGCTGGGATGAGATGGAAGA-3’
*mTOR*	5’-TTTGGACGGTGTGGAACTTG-3’	5’-CATCTGGGCCTCCAGTTAC-3’
*GSK-3*β	5’-CTGCACCTTCTTTCCAGTGA-3’	5’-GCATTGGTGCAGACAAGATG-3’
*GAPDH*	5’-GGGAAACTGTGGCGTGAT-3’	5’-GAGTGGGTGTCGCTGTTGA-3’
*P53*	5’-CTGAGGTTGGCTCTGACTGT-3’	5’-GCTGTTCCGTCCCAGTAGAT-3’
*MYC*	5’-ACACATCAGCACAACTACGC-3’	5’-CCTCTTGACATTCTCCTCGGT-3’
*EGFR*	5’-TCGATGAAGCCTACGTGATG-3’	5’-TTTGTGTTCCCGGACATAGT-3’
*GAPDH*	5’-GGGAAACTGTGGCGTGAT-3’	5’-GAGTGGGTGTCGCTGTTGA-3’


#### Assessment of ROS levels in hPDLFs using flow cytometry

Cells were collected by centrifugation in EP tubes and washed with phosphate-buffered saline (PBS). After centrifugation, the supernatant was discarded. 200 μL of DCFH-DA staining solution was added and mixed thoroughly. The cells were washed again with PBS and centrifuged at 1600 rpm for 5 min. After discarding the supernatant, 300 μL of PBS was added and mixed well. The cell suspension was then transferred to flow cytometry tubes and analysed using a flow cytometer (BD, America, FACSCanto). Results were obtained using a reactive oxygen species detection kit-Beyotime, China, S0033).

#### The expression of ROS-related proteins and PI3K/AKT-related proteins using Western blot (WB)

The expression of ROS-related proteins and *PI3K*/*AKT*-related proteins was analysed using Western blot. Samples were transferred into microcentrifuge tubes and lysed. The lysates were thoroughly homogenised and then centrifuged at 12,000 rpm at 4°C. Loading buffer was added to the resulting protein solution, which was subsequently denatured at 95°C and centrifuged. Total protein concentration was measured using a BCA assay kit (Beyotime, China, P0011). Following protein denaturation, SDS-PAGE electrophoresis was performed, and proteins were transferred to a membrane and blocked. The membrane was then incubated with primary antibodies at 4°C for 12 h. The primary antibodies and their dilutions were as follows: *TP53* (1:1000), *MYC* (1:1000), *EGFR* (1:1000), *SOD* (1:1000), *CAT* (1:1000), *Nrf2* (1:1000), *HO-1* (1:1000).

*PI3K* (1:1000), *PTEN* (1:1000), *AKT1* (1:1000), *AKT2* (1:1000), *mTOR* (1:1000), and *GSK-3*β (1:1000). After primary antibody incubation, the membrane was incubated with secondary antibodies at room temperature for 1 h. Protein signals were detected and visualised using the Immobilon Western Chemiluminescent HRP Substrate Kit (ABP Biosciences, China, FP302).

#### Functional rescue experiment on the anti-inflammatory effect of Tibetan medicine TRP through inhibition of the PI3K/Akt signalling pathway

The hPDLFs were divided into five groups: (1) blank control group; (2) LPS-treated group; (3) LPS + TRP group; (4) LPS + TRP + *PI3K* activator group (30 μM 740Y-P); (5) LPS + TRP + *PI3K* inhibitor group (50 μM LY294002). Subsequent experiments were conducted on these cell groups. The secretion levels of inflammatory factors interleukin-1 beta (IL-1β), interleukin-6 (IL-6), tumour necrosis factor-alpha (TNF-α), and matrix metalloproteinase-8 (MMP8) in the cell culture supernatants of each group were determined using the enzyme-linked immunosorbent assay (ELISA) method (SolarBio Science). Each sample was added to designated wells in a 96-well plate, which had been pre-filled with 40 µL of sample diluent provided with the kit. The plate was then incubated at 37°C for 1 h, followed by the addition of staining solution for 15 min before stopping the reaction. Absorbance was measured at 450 nm using an ELISA reader (Thermo Fisher Scientific, America, VL0000A), and concentrations were evaluated using a standard curve based on the absorbance readings. WB was used to detect the protein expression of *PI3K*, *Akt1*, and *Akt2* in the aforementioned five groups of cells, following the same procedure as previously described.

### Animal Experiments

#### Animals and ethical approval

Twenty-four healthy Sprague-Dawley rats (weighing 200–300 g) were purchased from Guangdong Medical Laboratory Animal Centre. All rats were housed under specific pathogen-free conditions at 25°C with a 12-h light/dark cycle and provided standard chow and water *ad libitum*. All animal experiments were conducted in accordance with guidelines approved by the Animal Care and Use Committee and complied with national regulations for experimental animal welfare and ethics.

#### Periodontitis rat model establishment

The periodontitis model was established according to previously described methods. Under sterile conditions, rats were anesthetised by intraperitoneal injection of 1% pentobarbital sodium (0.4 mL/100 g body weight). The maxillary region was exposed, and orthodontic ligature wire was placed around the cervical region of the bilateral maxillary second molars. Additionally, lipopolysaccharide from *Porphyromonas gingivalis* (10 μg/mL; InvivoGen, France, tlrl-pglps) was injected into the periodontal pockets every two days for a total of five injections. Subsequently, rats were randomly divided into four groups (n = 6 per group): (1) control group (no treatment); (2) model group (ligature + LPS); (3) saline group (ligature + LPS with saline irrigation of periodontal tissue, four times weekly); and (4) TRP group (ligature + LPS with 1.2% TRP irrigation of periodontal tissue, four times weekly). All animals were sacrificed after 2 months.

#### Western blotting analysis of rat periodontal tissues

Periodontal bone and soft tissue homogenates were transferred to microcentrifuge tubes and centrifuged at 12,000 rpm for 10 min at 4°C. The supernatant was collected, and protein concentration was determined using a BCA assay kit (Beyotime Biotechnology, Shanghai, China). Protein samples were mixed with loading buffer, denatured at 95°C for 10 min, and centrifuged. Denatured protein samples were separated by SDS-PAGE electrophoresis and transferred to 0.45 μm PVDF membranes (Millipore, MA, USA). Membranes were blocked with 5% non-fat milk for 1 h at room temperature. Primary antibodies were diluted 1:1000 and incubated with membranes overnight at 4°C. After washing with Tris-buffered saline containing 0.1% Tween 20 (Servicebio, China), membranes were incubated with secondary antibodies for 1 h at room temperature. Protein signals were detected using the Immobilon Western Chemiluminescent HRP Substrate Kit (Beyotime, Shanghai, China). Band intensities were quantified using ImageJ software (NIH, Bethesda, MD, USA) and normalised to *GAPDH* as an internal reference.w

#### Real-time quantitative polymerase chain reaction (qPCR)

Tissues were ground to powder in liquid nitrogen, and 1 mL TRIzol reagent was added for complete cell lysis. Chloroform was added, and the mixture was vigorously shaken. After centrifugation, the aqueous phase containing RNA was carefully extracted. The isolated RNA was subjected to reverse transcription. Target gene primers included IL-1β (120bp), IL-6 (221bp), TNF-α (100bp), RANKL (180bp), and OPG (150bp).

Amplification was performed in duplicate using Brilliant QPCR Master Mix (TransGen Biotech, China, AE301-03) and AceQ qPCR SYBR Green Master Mix (YEASEN, China, 11202ES08) with optimised primer/probe concentrations in a 25 μL reaction system. The reaction was performed using the ABI PRISM® 7500 Sequence Detection System (Applied Biosystems, USA, ABI-7500) with the following conditions: 95°C for 5 min; 40 cycles of 95°C for 15 s and 60°C for 32 s with fluorescence reading; melting curve analysis from 60°C to 95°C. Relative expression levels were calculated using the 2^−ΔΔCt method and normalised to β-actin as the internal reference gene. The forward and reverse primer sequences used are listed in Table 3.

**Table 3:** Sequences of primers used in the animal experiments

Gene symbol	5′-forward primer-3′	5′-reverse primer-3′
IL-1β	TGATGACGACCTGCTAGTGT	TTGAGGTGGAGAGCTTTCAG
IL-6	CTGGAGTTCCGTTTCTACCT	TGGATGGTCTTGGTCCTTAG
TNF-α	CTACTCCCAGGTTCTCTTCAA	GCTGACTTTCTCCTGGTATGA
RANKL	CCAAGTTCGCATAACCTGAT	CCCAAAGTACGTCGCATCTT
OPG	CAGCCCAGACGAGATTGAGA	TACATCAGGCCCTTCAAGGT
β-actin	AGGGAAATCGTGCGTGACAT	GAACCGCTCATTGCCGATAG


#### Microcomputed tomography (micro-CT) scanning

To assess bone defects, rat maxillae were analysed using a high-resolution micro-CT scanner (Bruker, Germany). Excised maxillae were wrapped in moistened gauze and placed on an appropriately sized sample bed. Following the manufacturer’s instructions, initial scanning and reconstruction tests were performed to correct beam hardening effects and set optimal contrast ranges. Scanning parameters were set at 80 kV voltage, 313 μA current, and 17.4 μm resolution. After scanning, all projection images were reconstructed using NRecon software to generate cross-sectional images. DataViewer software was used for three-axis alignment of coronal, sagittal, and transverse sections to ensure accuracy and consistency. Three-dimensional (3D) images were generated and analysed according to software instructions. The alveolar bone region of the second molar (from root bifurcation to apex, cross-sectional dimensions 2.8 mm × 3 mm) was selected as the region of interest. CTAn software was used for morphological and density analysis. CTVox software was used to reconstruct and display 3D dynamic images. Bone volume/tissue volume ratio (BV/TV) was calculated as a key indicator of bone formation. The distance from the alveolar bone crest to the cementoenamel junction (ABC-CEJ) was measured to evaluate alveolar bone health status.

#### Hematoxylin-eosin (H&E) and TRAP staining

For histological analysis, rat maxillary alveolar bone specimens were fixed in 4% paraformaldehyde for 24 h, decalcified in 15% EDTA for 4 weeks, dehydrated through graded ethanol solutions, cleared with xylene (Sinopharm Chemical Reagent Co., China), and embedded in paraffin. Sections of 5 μm thickness were prepared. Sections were stained with hematoxylin and eosin (H&E), dehydrated with ethanol and xylene, and mounted with neutral balsam (Sinopharm Chemical Reagent Co., Ltd., China). Morphological changes in periodontal tissues were observed under an upright optical microscope (Olympus, Japan), with nuclei appearing blue and cytoplasm appearing red.

Subsequently, osteoclasts were stained with tartrate-resistant acid phosphatase (TRAP) staining kit (Servicebio, China). Under microscopy, osteoclasts displayed wine-red cytoplasm with light blue nuclei. TRAP-positive cells were counted on the alveolar septum surface between the first and second molars. Osteoclasts were identified as TRAP-positive multinucleated cells containing two or more nuclei that were in direct contact with the bone surface. The number of osteoclasts in the mesial and distal regions of each sample was summed, and the average value for each group was calculated.

## RESULTS

### Results of Network Pharmacology Analysis Screening and Target Prediction of TRP Compounds

**Fig 1a to e:**
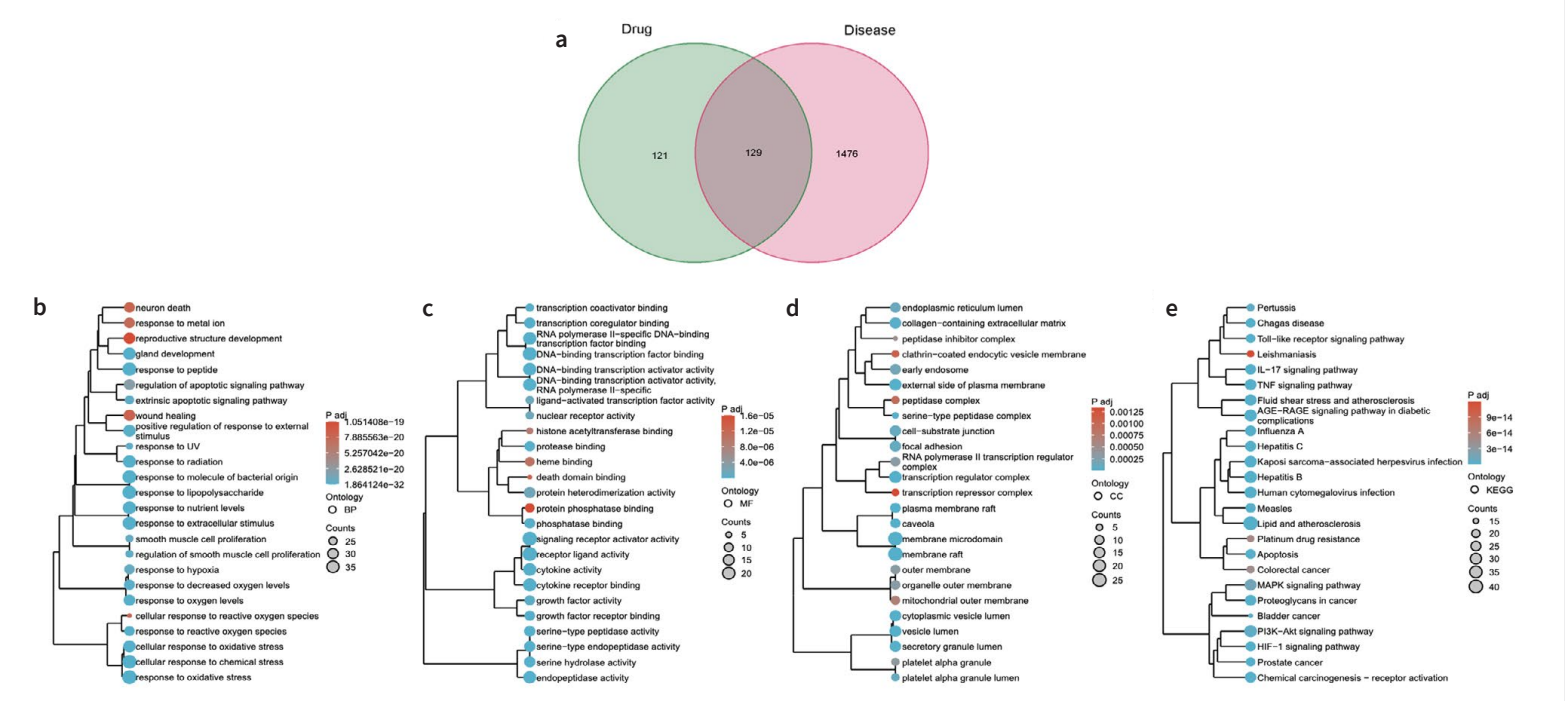
Network pharmacology analysis of Triphala’s therapeutic targets for periodontitis. (a) Venn diagram showing the intersection between Triphala drug targets and periodontitis-related genes, identifying 129 potential therapeutic targets of Triphala for treating periodontitis. (b) Significantly enriched gene ontology (GO) biological process terms of the potential drug target genes. (c) Significantly enriched GO molecular function terms. (d) Significantly enriched GO cellular component terms. (e) Significantly enriched KEGG pathways of the potential drug target genes.

Based on the TCMSP and TCM databases, 32 small molecules of the main herbs in TRP were screened (Table 2). Through target prediction of the small molecule compounds, 250 drug targets were obtained. By querying four databases, namely DisGeNET (https://www.disgenet.org/home/), GeneCards (https://www.genecards.org/), OMIM (https://www.omim.org/), and PharmGkb (https://www.pharmgkb.org/), a total of 1,605 periodontitis-related genes were identified. By taking the intersection of TRP drug targets and periodontitis genes, 129 potential targets of TRP for treating periodontitis were obtained (Fig 1a).

**Table 2:** Small molecules of TRP’s main herbal components. The table presents the 32 small molecules identified from the main herbal components of TRP, namely *Terminalia chebula* (TC), *Terminalia bellirica* (TB), and *Phyllanthus emblica* (PE), based on the Traditional Chinese Medicine Systems Pharmacology Database (TCMSP) and TCM Database@Taiwan. The table includes the compound name, molecule ID, oral bioavailability (OB) percentage, drug-likeness (DL) score, and the corresponding herb source for each small molecule. The OB and DL values were used as criteria for screening the main small molecule drug targets of TRP, with thresholds of OB ≥ 30% and DL ≥ 0.18

No.	Compound	Mol ID	OB (%)	DL	Herb
1	ellagic acid	MOL001002	43.06	0.43	TC,TB,PE
2	gallic acid	MOL000513	31.69	0.04	TC,TB,PE
3	corilagin	MOL005079	3.01	0.44	TC,TB
4	chebulic acid	MOL006826	72	0.32	TC, PE
5	7-Dehydrosigmasterol	MOL006376	37.42	0.75	TC
6	ellipticine	MOL009135	30.82	0.28	TC
7	Peraksine	MOL009136	82.58	0.78	TC
8	(R)-(6-methoxy-4-quinolyl)-[(2R,4R,5S)-5-vinylquinuclidin-2-yl]	MOL009137	55.88	0.4	TC
9	Cheilanthifoline	MOL009149	46.51	0.72	TC
10	quinic acid	MOL003069	55.92	0.06	TC
11	Teresautalic acid	MOL009138	41.42	0.09	TC
12	chebulinic acid	MOL009144	33.48	0.13	TC
13	Sennoside E_qt	MOL002276	50.69	0.61	TC
14	methyl gallate	MOL001906	30.91	0.05	TB
15	ethyl gallate	MOL001907	25.61	0.06	TB
16	salicin	MOL012219	7.15	0.16	TB
17	beta-sitosterol	MOL000358	36.91	0.75	PE
18	kaempferol	MOL000422	41.88	0.24	PE
19	(+)-catechin	MOL000492	54.83	0.24	PE
20	digallate	MOL000569	61.85	0.26	PE
21	leucodelphinidin	MOL005983	43.45	0.31	PE
22	luteolin	MOL000006	36.16	0.25	PE
23	mucic acid 1,4-lactone 2-0-gallate	MOL006793	49.56	0.31	PE
24	mucic acid 1,4-lactone 5-0-gallate	MOL006796	52.26	0.27	PE
25	(-)-epigalloca techin-3-gallate	MOL006821	55.09	0.77	PE
26	quercetin	MOL006799	46.43	0.28	PE
27	(2S,3R,3aS,4 R,4’S,5’R,6S,7aR)-3,4,4’-trihydroxy-3, 5’-bis(hydroxymethyl)spir o[3a,4,5,6,7,7a-hexahydrobenzofuran-2,2’-tetrahydropyran]-6-carboxylic acid	MOL006799	48.46	0.31	PE
28	phyllaemblic acid methylester	MOL006801	43.09	0.73	PE
29	phyllaemblicin A	MOL006802	45.63	0.77	PE
30	phyllanemblinin A	MOL006806	72.44	0.33	PE
31	Phyllanthin	MOL006812	33.31	0.42	PE
32	α-amyrin	MOL006824	39.51	0.76	PE


#### Functional enrichment analysis

Functional enrichment analysis was performed on the targets of TRP for treating periodontitis using clusterProfiler. As shown in Figure 1b, the 129 intersection genes are involved in biological processes such as response to hypoxia, response to decreased oxygen levels, response to oxygen levels, cellular response to oxidative stress, and response to oxidative stress. Figure 1c demonstrates that these intersection genes participate in molecular functions like signalling receptor activator activity, receptor ligand activity, cytokine activity, and cytokine receptor binding. Figure 1d reveals that these intersection genes are associated with cellular components such as focal adhesion, cell-substrate junction, and vesicle lumen. Figure 1e indicates that these intersection genes are involved in signalling pathways like *PI3K-AKT*, TNF, and MAPK.

#### Network construction and analysis

**Fig 2a and b:**
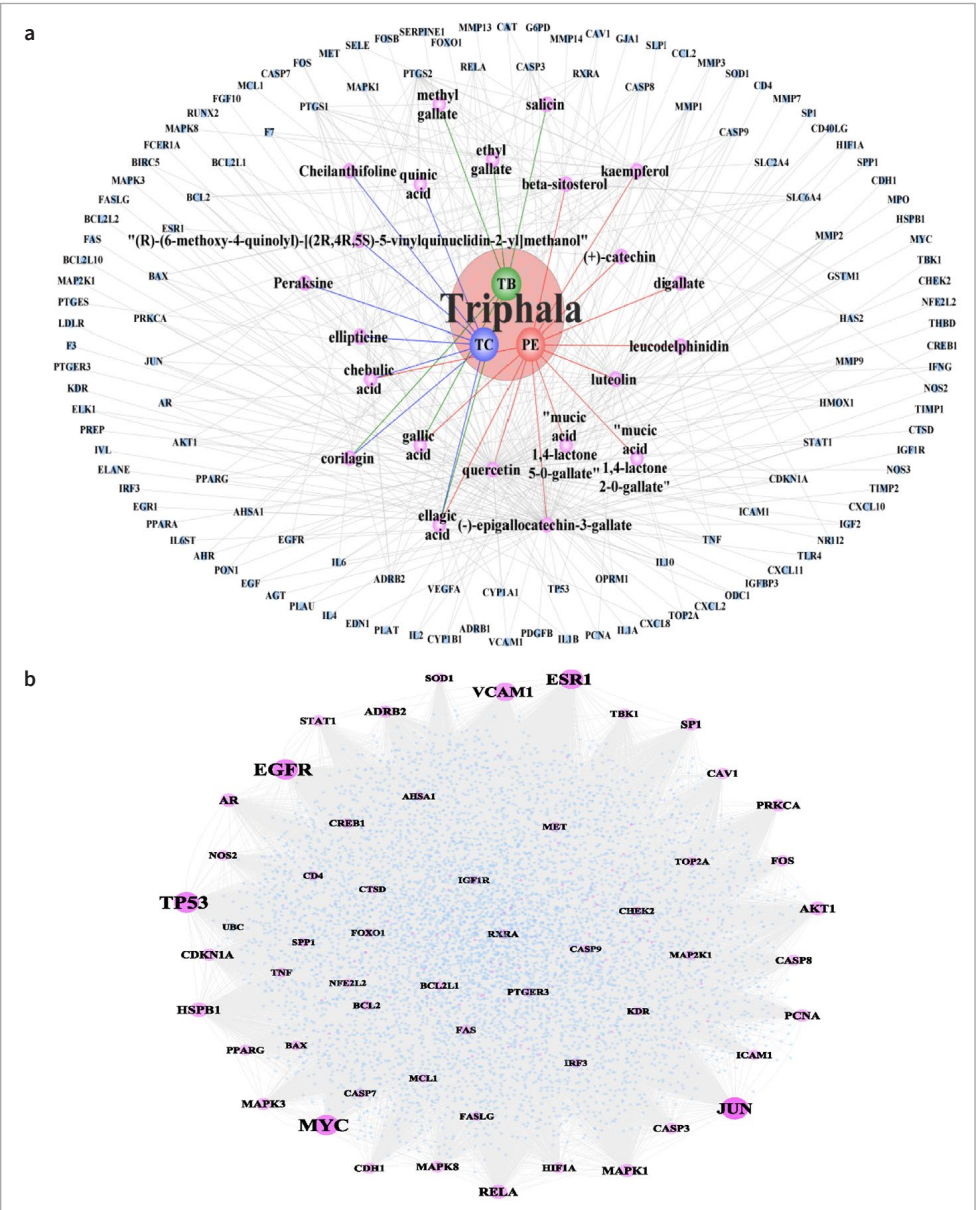
Network construction of Triphala-periodontitis interactions. (a) Triphala small molecule-periodontitis target network. The central pink node represents the Triphala compound formula, with three small circular nodes within representing the three constituent herbal components (*Terminalia chebula*, *Terminalia bellirica*, and *Phyllanthus emblica*). Purple nodes represent the active compounds derived from these three herbal components, and blue nodes represent the periodontitis-related genes targeted by these compounds. (b) Protein–protein interaction (PPI) network constructed based on the 129 potential drug targets of Triphala for treating periodontitis.

Subsequently, the small molecules corresponding to the drug targets of TRP for treating periodontitis were extracted, and the TRP drug target network was constructed using Cytoscape software (Fig 2a). This network contains a total of 151 nodes and 302 edges. Next, the protein interaction pairs of TRP’s drug targets for treating periodontitis were extracted, yielding 16,480 interaction pairs. These interaction pairs were used to construct a drug target protein interaction network, which comprises 6,449 nodes and 16,480 edges (Fig 2b). Topological analysis of the network was performed, and the top 30 genes were selected based on their degree in descending order (Table 4). The top 10 nodes in this PPI network (Fig 2b) are *JUN*, *TP53*, *MYC*, *EGFR*, *ESR1*, *MDM2*, *VCAM1*, *HSPB1*, *RELA*, and *AKT1*.

**Table 4:** Topological properties of the protein–protein interaction network for TRP’s drug targets in treating periodontitis. This table presents the topological properties of the top 30 nodes (genes) in the protein–protein interaction (PPI) network constructed for TRP’s drug targets in treating periodontitis. The nodes are ranked in descending order based on their degree centrality. The table includes the gene name, label (indicating its role as a drug target), degree centrality, average shortest path length, betweenness centrality, closeness centrality, clustering coefficient, and topological coefficient for each node

Name	Label	Degree	Average shortest pathlength	Between ness centralit y	Closeness central ity	Clustering coeffici ent	Topologi cal coeffici ent
*JUN*	drugtar	1528	2.099411	0.223448	0.4763	0.00211	0.00217
	get				24	1	9
*TP53*	drugtar	1112	2.051179	0.170916	0.4875	0.00413	0.00265
	get				25	2	9
*MYC*	drugtar	1048	2.200682	0.160383	0.4544	0.00258	0.00260
	get				05	1	6
*EGFR*	drugtar	1035	2.210298	0.160824	0.4524	0.00267	0.00272
	get				28	8	1
*ESR1*	drugtar	847	2.120037	0.09927	0.4716	0.00638	0.00331
	get				9	5	
*VCAM*	drugtar	678	2.66889	0.053649	0.3746	6.15E-0	0.00581
*1*	get				88	4	2
*HSPB*	drugtar	443	2.345378	0.064295	0.4263	0.00523	0.00461
*1*	get				7	9	6
*RELA*	drugtar	413	2.326923	0.042611	0.4297	0.01254	0.00584
	get				52	2	3
*AKT1*	drugtar	386	2.459522	0.039774	0.4065	0.00901	0.00589
	get				83	3	5
*AR*	drugtar	369	2.325837	0.030866	0.4299	0.01489	0.00651
	get				53	6	
*MAPK*	drugtar	346	2.222395	0.036891	0.4499	0.01960	0.00738
*1*	get				65	2	8
*PCNA*	drugtar	319	2.443083	0.036618	0.4093	0.00641	0.00571
	get				19		1
*ADRB*	drugtar	296	2.900744	0.026089	0.3447	5.11E-0	0.02060
*2*	get				39	4	3
*CDKN*	drugtar	295	2.460453	0.031297	0.4064	0.01018	0.00688
*1A*	get				29	3	2
*SP1*	drugtar	292	2.353443	0.026711	0.4249	0.02024	0.00801
	get				09		9
*MAPK*	drugtar	284	2.354373	0.027156	0.4247	0.01584	0.00762
*3*	get				41	3	7
*PRKC*	drugtar	278	2.57987	0.033807	0.3876	0.00436	0.00632
*A*	get				16		8
*MAPK*	drugtar	236	2.297457	0.022923	0.4352	0.02586	0.00934
*8*	get				64	4	6
*FOS*	drugtar	236	2.49116	0.02259	0.4014	0.01235	0.00806
	get				19		3
*CAV1*	drugtar	220	2.547146	0.023731	0.3925	0.01462	0.00922
	get				96	7	2
*STAT*	drugtar	219	2.364764	0.022194	0.4228	0.01878	0.00959
*1*	get				75	7	5
*CASP*	drugtar	215	2.553505	0.016872	0.3916	0.02281	0.00984
*3*	get				19	5	6
*CASP*	drugtar	214	2.591967	0.016378	0.3858	0.01999	0.00993
*8*	get				07		7
*HIF1A*	drugtar	185	2.406948	0.017045	0.4154	0.0293	0.01152
	get				64		
*CDH1*	drugtar	178	2.70549	0.01695	0.3696	0.01011	0.01065
	get				19	4	3
*TBK1*	drugtar	168	2.691532	0.015186	0.3715	0.01327	0.01054
	get				36	6	4
*PPAR*	drugtar	166	2.540167	0.010499	0.3936	0.0292	0.01213
*G*	get				75		4
*NOS2*	drugtar	164	2.859491	0.008255	0.3497	0.00503	0.01374
	get				13	1	
*SOD1*	drugtar	162	2.947736	0.019145	0.3392	0.00212	0.01901
*ICAM*	drugtar	159	2.81188	0.008361	0.3556	0.00465	0.01062
*1*	get				34	5	8


#### GO and KEGG pathway analyses

**Fig 3:**
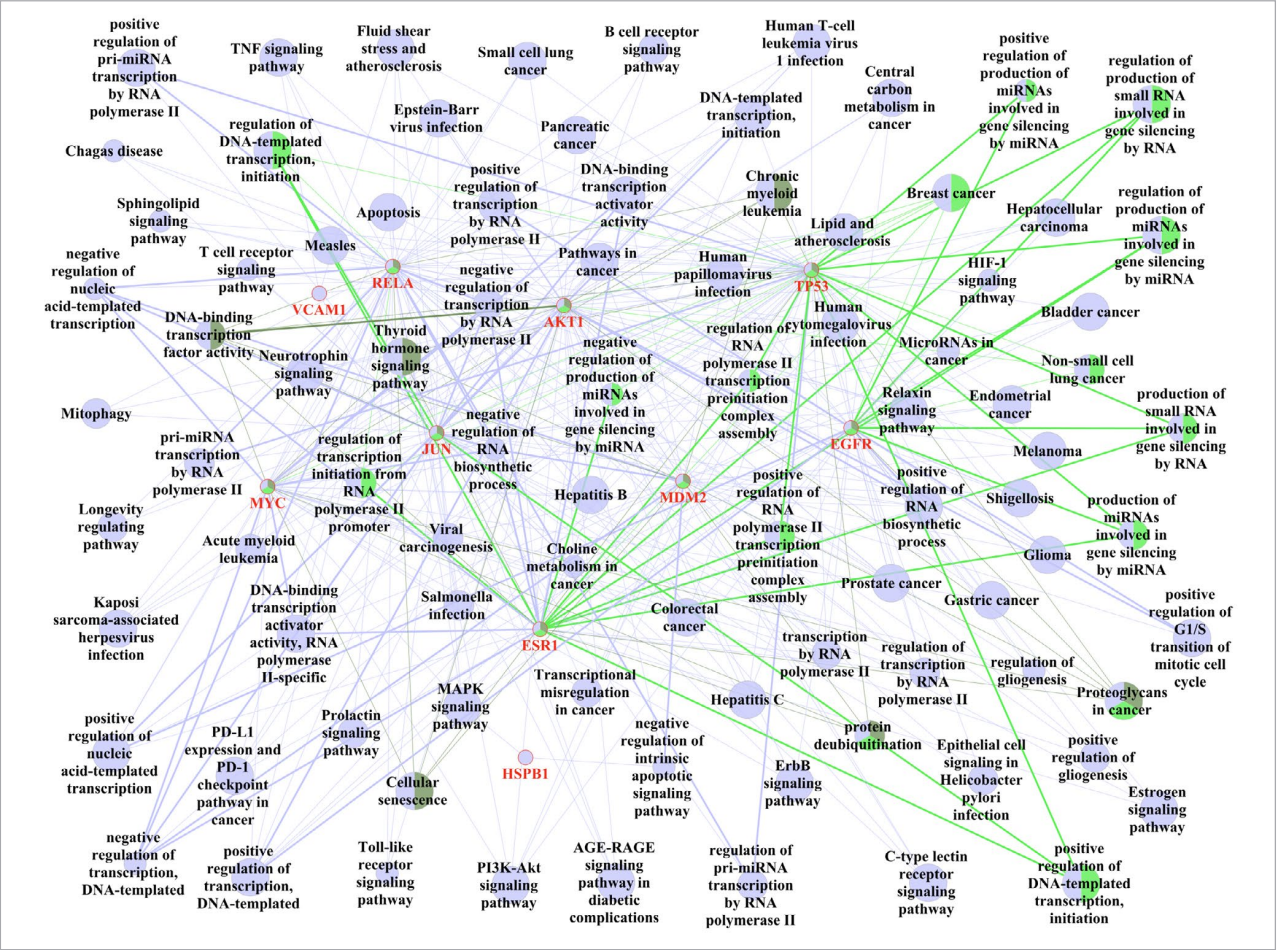
Functional enrichment analysis of top-hub genes. Interaction network of functions enriched by the top 10 hub target genes extracted from the PPI network, analysed using ClueGO and CluePedia (P adjust ≤ 0.05).

Afterwards, GO biological process and KEGG pathway analyses were conducted using the Cytoscape plugins ClueGO and CluePedia, with a p.adjust value less than or equal to 0.05. The top 10 hub genes-enriched functions interaction network was constructed (Fig 3). The main signalling pathways of TRP’s top 10 drug targets include *PI3K-Akt* signalling pathway, Toll-like receptor signalling pathway, MAPK signalling pathway, ErbB signalling pathway, and others (Fig 3).

#### Cell toxicity assessment using the CCK-8 method

**Fig 4a to g:**
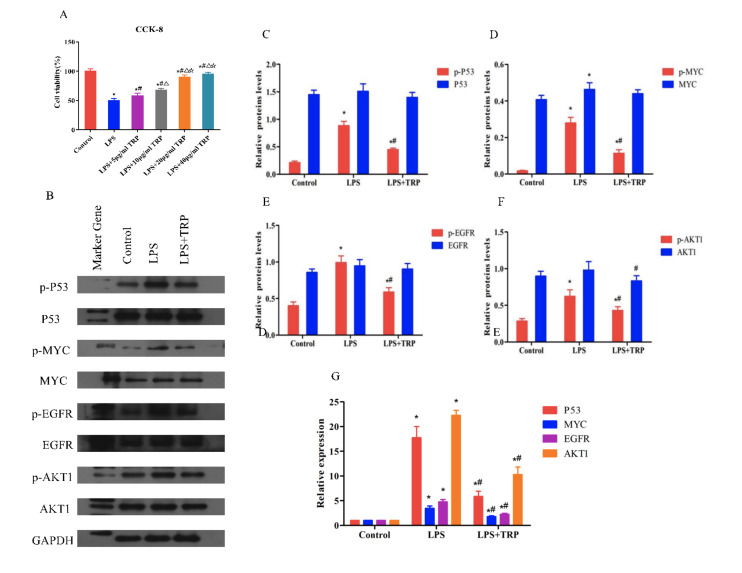
Optimal concentration of TRP and its effects on LPS-Induced cells. (a) Cytotoxicity of various concentrations of TRP in periodontal ligament fibroblasts over 24 h. Data are presented as mean ± standard error (n = 6 independent experiments); statistical analysis was performed using one-way ANOVA followed by Tukey’s post-hoc test. P < 0.05 compared to the control group; #P < 0.05 compared to the LPS group; △P < 0.05 compared to the LPS + 5 μg/ml TRP group; P < 0.05 compared to the LPS + 10 μg/ml TRP group. (b) Representative Western blot images showing protein expression levels of *P53*, *MYC*, *EGFR*, and *AKT1* in periodontal ligament fibroblasts treated with LPS and TRP. (c to f) Quantitative analysis of protein expression of *P53* (c), *MYC* (d), *EGFR* (e), and *AKT1* (f) normalised to *GAPDH*, as shown by Western Blot. Data are presented as mean ± standard error (n = 6 independent experiments); statistical analysis was performed using one-way ANOVA followed by Tukey’s post-hoc test. P < 0.05 compared to the control group; #P < 0.05 compared to the LPS group. (g) Relative mRNA expression levels of *P53*, *MYC*, *EGFR*, and *AKT1* in periodontal ligament fibroblasts treated with LPS and TRP, analysed by qRT-PCR and normalised to *GAPDH*. Data are presented as mean ± standard error (n = 6 independent experiments); statistical analysis was performed using one-way ANOVA followed by Tukey’s post-hoc test.** *P < 0.05 compared to the control group; #P < 0.05 compared to the LPS group.

hPDLFs were subcultured as shown in Figure 4a. Pure cell control group, cells treated with 1 μg/ml LPS, and cells treated with 1 μg/ml LPS for 24 h were then exposed to four concentrations of TRP (5 μg/ml, 10 μg/ml, 20 μg/ml, 40 μg/ml). In the LPS group, cell viability was inhibited by nearly half (P < 0.01). The LPS+5 μg/ml TRP group showed a trend of reduced inhibition of cell viability compared to the LPS group (P < 0.01). The LPS+10 μg/ml TRP group demonstrated alleviated inhibition and increased cell viability compared to the LPS group (P < 0.01), and higher cell viability than the LPS+5 μg/ml TRP group (P < 0.01). The LPS+20 μg/ml TRP group showed significantly increased cell viability compared to the LPS group (P < 0.01), and a more pronounced increase compared to both LPS+5 μg/ml and LPS+10 μg/ml TRP groups (P < 0.01). The LPS+40 μg/ml TRP group showed no statistical difference from the LPS+20 μg/ml TRP group, but was statistically different from both LPS+5 μg/ml and LPS+10 μg/ml TRP groups (P < 0.01). A subsequent CCK-8 assay using 20 μg/ml TRP revealed no significant inhibition of cell viability at this concentration. This experiment established the optimal TRP concentration for subsequent experiments.

#### Expression analysis of hub genes identified through network pharmacology

WB analysis was used to determine the protein expression trends of four hub genes – *P53*, *MYC*, *EGFR*, and *AKT1* – in LPS-induced hPDLFs treated with TRP, as shown in Figure 4b. After LPS addition, the expression of *P53* (Fig 4c), *MYC* (Fig 4d), *EGFR* (Fig 4e), and *AKT1* (Fig 4f) was upregulated in the inflammatory environment compared to the control group (P < 0.01). In the LPS+TRP group, the expression of *P53* (Fig 4c), *MYC* (Fig 4d), *EGFR* (Fig 4e), and *AKT1* (Fig 4f) was downregulated compared to the LPS group (P < 0.01), but slightly increased compared to the control group. To further examine the gene expression of these four hub genes under inflammatory conditions with TRP treatment, PCR analysis was conducted on *P53*, *MYC*, *EGFR*, and *AKT1* genes. As shown in Figure 4 g, the expression levels of *TP53*, *MYC*, *EGFR*, and *AKT1* were upregulated compared to the blank control group (P < 0.01). In the LPS+TRP group, the expression of these hub genes was downregulated to varying degrees compared to the LPS group (P < 0.01), but slightly upregulated compared to the control group. These results were consistent with the Western blot findings.

#### Experiment on TRP’s inhibition of LPS-induced ROS in hPDLFs

**Fig 5a to d:**
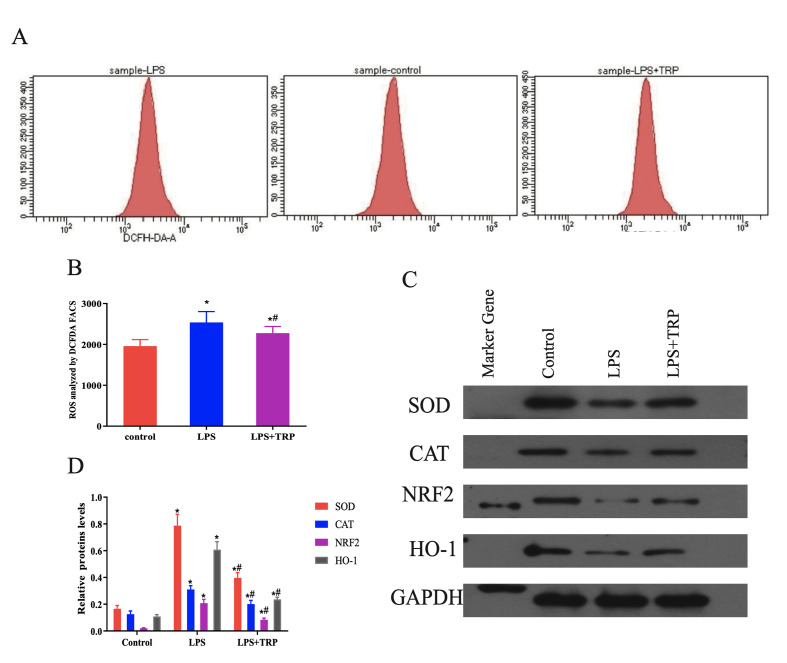
TRP alleviates LPS-induced oxidative stress at the cellular level. (a) Representative flow cytometry histograms showing analysis of cellular ROS levels using DCFH-DA dye in periodontal ligament fibroblasts treated with Control, LPS, or LPS+TRP. (b) Quantitative analysis of flow cytometry results showing the mean fluorescence intensity of ROS. Data are presented as mean ± standard error (n = 6 independent experiments); statistical analysis was performed using one-way ANOVA followed by Tukey’s post-hoc test. *P < 0.05 compared to the control group; #P < 0.05 compared to the LPS group. (c) Representative Western blot images showing protein expression levels of SOD, CAT, NRF2, and HO-1 in periodontal fibroblasts treated with LPS and TRP. (d) Quantitative analysis of protein expression normalised to *GAPDH* as shown by Western Blot. Data are presented as mean ± standard error (n = 6 independent experiments); statistical analysis was performed using one-way ANOVA followed by Tukey’s post-hoc test. *P < 0.05 compared to the control group; #P < 0.05 compared to the LPS group.

hPDLFs were treated with LPS alone or LPS+TRP for 24 h and compared to an untreated control group to investigate TRP’s mitigating effect on LPS-induced oxidative stress. Using a reactive oxygen species detection kit (Fig 5a and b), the LPS group showed a significant increase in ROS compared to the control group (P < 0.01). This effect was notably reduced after TRP treatment (P < 0.01). Additionally, WB analysis was used to detect the protein expression of SOD, CAT, Nrf2, and HO-1 in these three groups. As shown in Figure 5c and d, compared to the control group, the LPS group exhibited an increasing trend in the expression of SOD, CAT, Nrf2, and HO-1 (P < 0.01). In the LPS+TRP group, the expression of these ROS-related proteins showed a decreasing trend compared to the LPS group (P < 0.01).

#### Verification of TRP’s anti-inflammatory effect on LPS-induced inflammation in hPDLFs through regulation of the PI3K-Akt signalling pathway

**Fig 6a to c:**
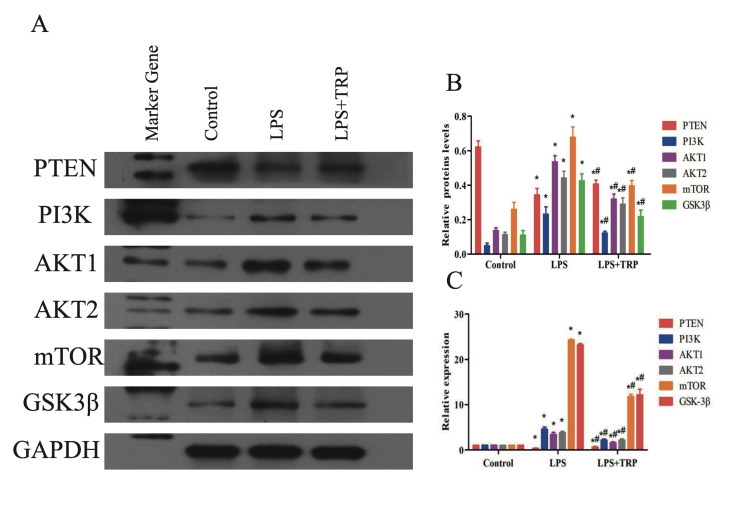
Effects of TRP on the *PI3K*/*AKT* signalling pathway. (a) Representative Western blot images showing protein expression levels of *PTEN*, *PI3K*, *AKT1*, *AKT2*, *mTOR*, and *GSK-3*β in periodontal ligament fibroblasts treated with LPS and TRP. (b) Quantitative analysis of protein expression normalised to *GAPDH* as shown by Western Blot. Data are presented as mean ± standard error (n = 6 independent experiments); statistical analysis was performed using one-way ANOVA followed by Tukey’s post-hoc test. p < 0.05 compared to the control group; #P < 0.05 compared to the LPS group. (c) Relative mRNA expression levels of *PI3K*/AKT signalling pathway-related proteins*, analysed by qRT-PCR and normalised to GAPDH**. Data are presented as mean ± standard error (n = 6 independent experiments); statistical analysis was performed using one-way ANOVA followed by Tukey’s post-hoc test. *P < 0.05 compared to the control group; #P < 0.05 compared to the LPS group.

Network pharmacology results suggest that TRP’s impact on periodontitis is primarily achieved through the regulation of certain signalling pathways. This experiment focuses on investigating TRP’s effect on the *PI3K-AKT* signalling pathway. PCR was used to detect the expression levels of upstream genes (*PI3K*, *PTEN*) and downstream genes (*AKT1*, *AKT2*, *mTOR*, *GSK-3*β) in the PI3K-Akt signalling pathway. As shown in Figure 6c, *PI3K*, *AKT1*, *AKT2*, *mTOR*, and *GSK-3*β were upregulated in the LPS group compared to the control group (P < 0.01), while *PTEN* was downregulated (P < 0.01). Inthe LPS+TRP group, *PI3K*, *AKT1*, *AKT2*, *mTOR*, and *GSK-3*β showed decreased expression compared to the LPS group (P < 0.01), while *PTEN* expression was upregulated (P < 0.01). Subsequently, WB experiments were conducted to further verify TRP’s effect on the *PI3K*-AKT signalling pathway at the protein level. As shown in Figures 6a and b, protein expression of *PI3K*, *AKT1*, *AKT2*, *mTOR*, and *GSK-3*β in the LPS group was increased compared to the control group (P < 0.01), while *PTEN* protein expression was reduced (P < 0.01). In the LPS+TRP group, protein expression of *PI3K*, *AKT1*, *AKT2*, *mTOR*, and *GSK-3*β was relatively lower compared to the LPS group (P < 0.01) but higher than the control group (P < 0.01). *PTEN* protein expression in the LPS + TRP group was lower than in the LPS group (P < 0.01) but higher than in the control group (P < 0.01).

#### Functional rescue experiment confirming the involvement of the PI3K/Akt signaling pathway in TRP-mediated anti-inflammatory effects

**Fig 7a to h:**
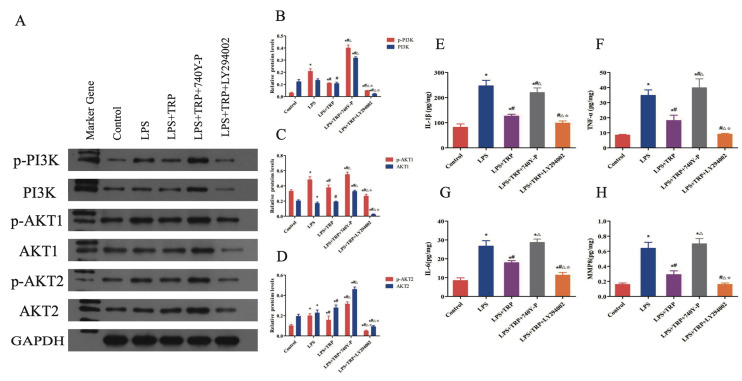
Anti-inflammatory Effects of TRP on the *PI3K*/AKT signalling pathway. (a) Representative Western blot images showing changes in phosphorylated and total protein levels of *PI3K*, *AKT1* and *AKT2* in periodontal ligament fibroblasts treated with control, LPS, LPS+TRP, LPS+TRP+740Y-P (*PI3K* activator), or LPS+TRP+LY294002 (*PI3K* inhibitor). (b to d) Quantitative analysis of phosphorylated protein changes of *PI3K* (b), *AKT1* (c) and *AKT2* (d) normalised to their respective total protein levels. Data are presented as mean ± standard error (n = 6 independent experiments); statistical analysis was performed using one-way ANOVA followed by Tukey’s post-hoc test. *P < 0.05 compared to the control group; #P < 0.05 compared to the LPS group; △P < 0.05 compared to the LPS + TRP + 740Y-P group; P < 0.05 compared to the LPS + TRP + LY294002 group. (e to h) Changes in the secretion levels of inflammatory cytokines IL-1β (E), TNF-α (F), IL-6 (G), and MMP8 (h) in cell culture supernatants measured by ELISA. Data are presented as mean ± standard error (n = 6 independent experiments); statistical analysis was performed using one-way ANOVA followed by Tukey’s post-hoc test. *P < 0.05 compared to the control group; #P < 0.05 compared to the LPS group; △P < 0.05 compared to the LPS + TRP + 740Y-P group; P < 0.05 compared to the LPS + TRP + LY294002 group.

hPDLFs were divided into five groups: control, LPS, LPS+TRP, LPS+TRP+*PI3K* activator (30μM 740Y-P), and LPS+TRP+*PI3K* inhibitor (50μM LY294002). However, there was no statistically significant difference compared to the control group. WB analysis was used to detect the protein expression levels of key PI3K/Akt signalling pathway proteins *PI3K*, *Akt1*, and *Akt2*, as shown in Figure 7a to d. Results indicated that phosphorylated *PI3K*, *Akt1*, and *Akt2* protein expression in the LPS group showed an upward trend compared to the control (P < 0.01). In the LPS+TRP group, their protein expression levels were lower than those of the LPS group (P < 0.01) but higher than those of the control (P < 0.01). In the LPS+TRP+740Y-P group, phosphorylated *PI3K*, *Akt1*, and *Akt2* proteins were significantly upregulated compared to the LPS+TRP group (P < 0.01) and showed higher protein expression than both the LPS and control groups (P < 0.01). The LPS+TRP+LY294002 group showed a downward trend compared to the LPS+TRP+740Y-P, LPS+TRP, and LPS groups (P < 0.01), with a slight upregulation compared to the control (P < 0.01). As shown in Figure 7e to h, IL-1β, IL-6, TNF-α, and MMP8 levels were significantly elevated in the LPS group compared to the control (P < 0.01). In the LPS+TRP group, these inflammatory factors were markedly reduced compared to the LPS group (P < 0.01), but slightly increased compared to the control (P < 0.01). In the LPS+TRP+740Y-P group, the secretion levels of IL-1β, IL-6, TNF-α, and MMP8 increased compared to the LPS+TRP group (P < 0.01), slightly decreased compared to the LPS group (P < 0.01), and significantly increased compared to the control (p<0.01). The expression of inflammatory factors in the LPS+TRP+LY294002 group was significantly lower than in the LPS+TRP+740Y-P group, slightly downregulated compared to the LPS+TRP group, and significantly downregulated compared to the LPS group compared to the control group; #P < 0.05 compared to the LPS group; △P < 0.05 compared to the LPS + TRP + 740Y-P group; P < 0.05 compared to the LPS + TRP + LY294002 group.

#### Detection of IL-1 -beta, IL-6, and TNF-alpha inflammatory cytokines in rat periodontal tissues

**Fig 8a to e:**
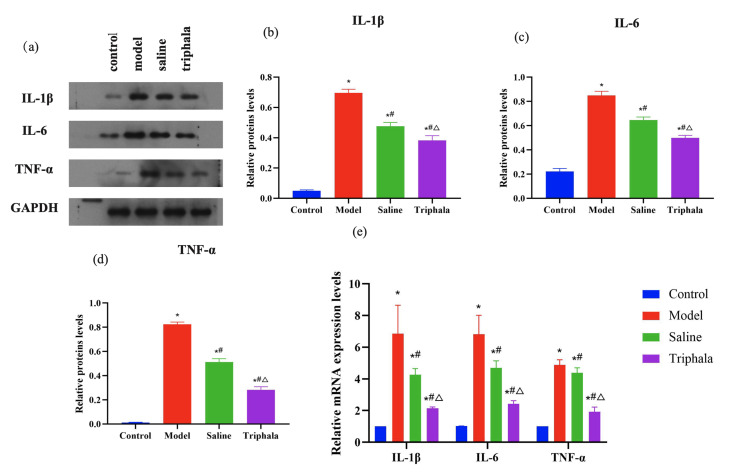
Expression of IL-1β, IL-6, and TNF-α in rat periodontal tissues. (a) Representative Western blot images showing analysis of IL-1β, IL-6, and TNF-α protein expression in periodontal tissues from control, model (ligature + LPS), saline (ligature + LPS + saline irrigation), and TRP (ligature + LPS + TRP irrigation) groups. (b) Quantification of IL-1β protein expression normalised to *GAPDH*. (c) Quantification of IL-6 protein expression normalised to *GAPDH*. (d) Quantification of TNF-α protein expression normalised to *GAPDH*. Data in panels b to d are presented as mean ± standard error (n = 6 animals per group); statistical analysis was performed using one-way ANOVA followed by Tukey’s post-hoc test. (e) Relative mRNA expression levels of IL-1β, IL-6, and TNF-α were determined by qPCR and normalised to β-actin. Data are presented as mean ± standard error (n = 6 animals per group); statistical analysis was performed using one-way ANOVA followed by Tukey’s post-hoc test. *P < 0.05 compared to the control group; #P < 0.05 compared to the model group; △P < 0.05 compared to the saline group.

IL-1β, IL-6, and TNF-α are three crucial inflammatory biomarkers that play pivotal roles in pro-inflammatory responses. Western blot analysis and quantification of band intensities (Fig 8a to d) revealed that the control group exhibited low protein expression levels of IL-1β, IL-6, and TNF-α. Compared to the control group, the model group showed significantly increased protein expression of these inflammatory cytokines (P < 0.05). The saline group demonstrated elevated expression of IL-1β, IL-6, and TNF-α compared to the control group (P < 0.05), but reduced expression compared to the model group (P < 0.05). The TRP group showed increased protein expression of these cytokines compared to the control group (P < 0.05), but decreased expression compared to the saline group (P < 0.05).

qPCR analysis of mRNA expression levels (Fig 8e) demonstrated that the control group exhibited relatively low expression of IL-1β, IL-6, and TNF-α inflammatory cytokines. Compared to the control group, the model group showed significantly elevated mRNA expression levels of IL-1β, IL-6, and TNF-α (P < 0.05). The saline group displayed a downward trend in the expression of these cytokines compared to the model group (P < 0.05), while showing upregulated expression compared to the control group (P < 0.05). The TRP group exhibited increased expression of IL-1β, IL-6, and TNF-α compared to the control group (P < 0.05), but decreased expression relative to the saline group (P < 0.05).

#### TRP inhibits periodontitis progression and promotes alveolar bone repair

**Fig 9a to c:**
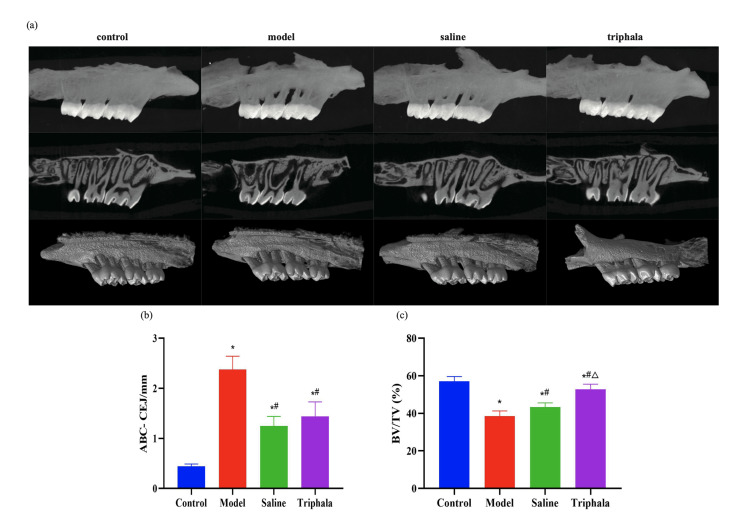
TRP protects against LPS-induced periodontal bone loss in rats. (a) Representative three-dimensional reconstructed micro-CT images showing alveolar bone defects around the maxillary first molar in control, model (ligature+LPS), saline (ligature+LPS+saline irrigation), and TRP (ligature+LPS+TRP irrigation) groups. (b) Bone volume fraction (BV/TV) measurements in each experimental group. (c) Distance from alveolar bone crest to cementoenamel junction (ABC-CEJ) measurements in each experimental group. Data in panels b and c are presented as mean ± standard error (n = 6 animals per group); statistical analysis was performed using one-way ANOVA followed by Tukey’s post-hoc test. *P < 0.05 compared to the control group; #P < 0.05 compared to the model group; △P < 0.05 compared to the saline group.

The primary manifestation of periodontitis is alveolar bone destruction and resorption. Micro-CT imaging analysis of the maxillary first molar was performed 2 months after periodontitis induction (Fig 9a and b). Analysis of the distance from the alveolar bone crest (ABC) to the cemento-enamel junction (CEJ) revealed that the control group showed no significant alveolar bone loss, with minimal ABL values. The model, saline, and TRP groups all exhibited varying degrees of alveolar bone resorption, with notably increased ABL values. Compared to the control group, the model group showed significantly increased ABL values (P < 0.05), indicating enhanced alveolar bone loss. The saline group demonstrated relatively increased ABL values compared to the control group (P < 0.05), but showed a downward trend compared to the model group (P < 0.05). The TRP group results showed that alveolar bone loss increased compared to the control group with elevated ABL values, but decreased relative to the saline group with slightly reduced ABL values (P > 0.05), and significantly decreased ABL values compared to the model group (P < 0.05).

Furthermore, bone volume fraction (BV/TV) results were consistent with the ABL findings (Fig 9c). Compared to the control group, the model group showed significantly reduced BV/TV (P < 0.05), the saline group showed relatively reduced BV/TV (P < 0.05), while the TRP group exhibited the smallest reduction (P < 0.05). Compared to the model group, the saline group showed attenuated BV/TV reduction with an increased ratio (P < 0.05), and the TRP group demonstrated an elevated BV/TV ratio (P < 0.05).

#### TRP modulates RANKL/OPG expression in periodontitis development

**Fig 10a to c:**
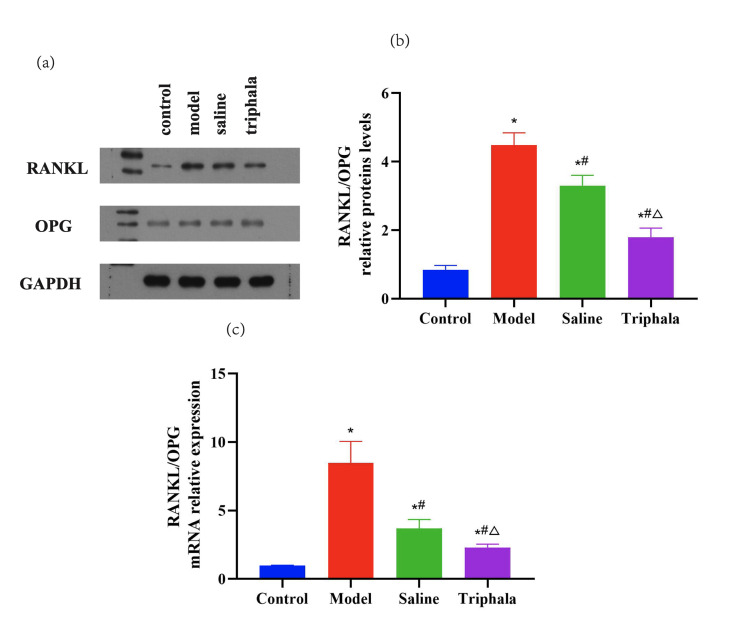
Expression of RANKL and OPG in rat periodontal bone tissue. (a) Representative Western blot images showing analysis of RANKL and OPG protein expression in periodontal bone tissues from control, model (ligature+LPS), and saline.

RANKL and OPG play crucial roles in osteoclastogenesis and bone formation, serving as key mediators in the initiation and progression of periodontitis. Western blot analysis (Fig 10a and b) revealed that the RANKL/OPG ratio was significantly increased in the model group compared to the control group (P < 0.05), indicating exacerbated alveolar bone resorption. The saline group showed an increased RANKL/OPG ratio compared to the control group (P < 0.05), but demonstrated a reduced ratio compared to the model group (P < 0.05). The TRP group exhibited an elevated RANKL/OPG ratio compared to the control group (P < 0.05), but showed a decreased ratio compared to the model group (P < 0.05), with a greater reduction magnitude than the saline group (P < 0.05). These findings suggest that TRP may inhibit bone resorption and promote bone repair through modulation of the RANKL/OPG axis, indicating its potential for periodontal tissue regeneration and bone protection.

qPCR analysis further validated these results (Table 5). RANKL expression was significantly elevated in the model group compared to the control group (8.115 ± 0.626 vs 1.000 ± 0.0001, P < 0.05). The saline group showed increased RANKL expression compared to the control group (3.653 ± 0.557, P < 0.05), but slightly decreased expression compared to the model group (P < 0.05). In the TRP group, RANKL expression was elevated compared to the control group (2.088 ± 0.171, P < 0.05), but significantly reduced compared to both the model and saline groups (P < 0.05).

**Table 5:** Quantitative real-time PCR analysis of RANKL and OPG mRNA expression in rat periodontal tissues.

Group	n	RANKL	OPG	RANKL_OPG
Control	6	1.000 ± 0.0001	1.002 ± 0.003	0.999 ± 0.003
model	6	8.115 ± 0.626*	0.978 ± 0.151	8.481 ± 1.56*
Saline	6	3.653 ± 0.557*#	0.999 ± 0.078	3.682 ± 0.668*#
TRP	6	2.088 ± 0.171*#△	0.916 ± 0.121	2.3 ± 0.242*#△
F		321.573	0.888	87.197
P		< 0.001	0.465	< 0.001
*P < 0.05 vs control; #P < 0.05 vs model; P < 0.05 vs saline				

OPG expression showed less pronounced differences among groups, with no statistically significant changes (P = 0.465). The control group showed OPG expression of 1.002 ± 0.003, the model group 0.978 ± 0.151, the saline group 0.999 ± 0.078, and the TRP group 0.916 ± 0.121.

The RANKL/OPG ratio, which reflects the balance between bone resorption and formation in molecular biology experiments, showed consistent trends (Fig 10c). The model group demonstrated a significantly increased RANKL/OPG ratio compared to the control group (8.481 ± 1.56 vs 0.999 ± 0.003, P < 0.05). Both the saline group (3.682 ± 0.668) and TRP group (2.3 ± 0.242) showed reduced RANKL/OPG ratios compared to the model group (P < 0.05), with the TRP group demonstrating a greater reduction magnitude.

### Effects of TRP on Periodontal Osteoclasts in Rats

**Fig 11:**
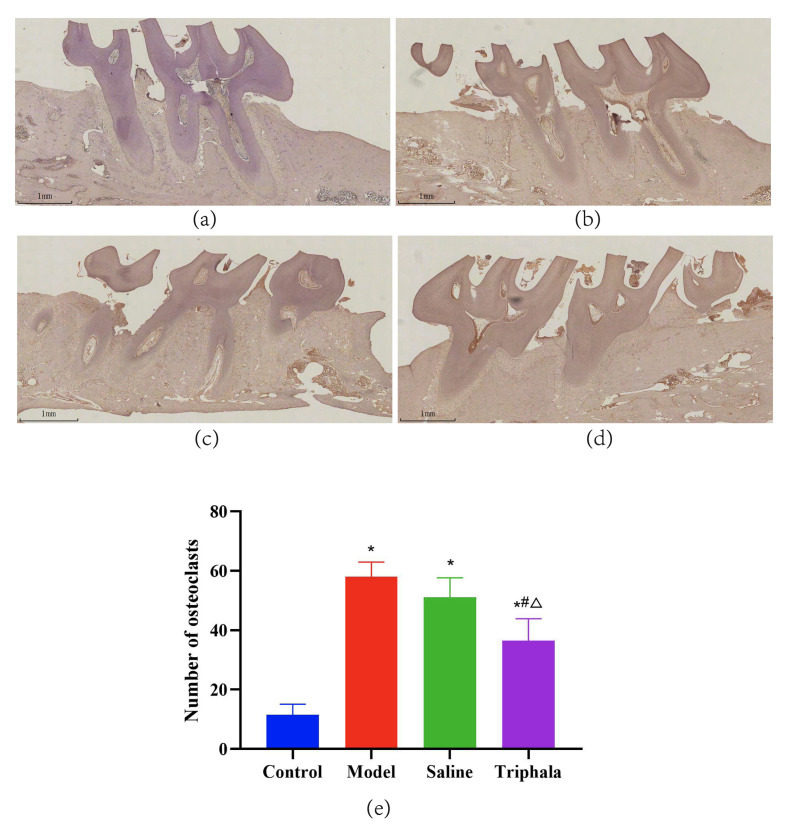
Visualisation of osteoclasts in rat periodontal tissues using tartrate-resistant acid phosphatase (TRAP) staining. (a) Representative TRAP-stained section from the control group showing minimal osteoclast presence. (b) Representative TRAP-stained section from the model group showing increased TRAP-positive osteoclasts (indicated by wine-red cytoplasm) along the alveolar bone surface. (c) Representative TRAP-stained section from the saline group. (d) Representative TRAP-stained section from the TRP group showing reduced osteoclast numbers. Scale bar information and magnification should be indicated in the images. (e) Quantification of TRAP-positive osteoclast numbers on the alveolar septum surface between the first and second molars in each group. Data are presented as mean ± standard error (n = 6 animals per group); statistical analysis was performed using one-way ANOVA followed by Tukey’s post-hoc test. *P < 0.05 compared to the control group; #P < 0.05 compared to the model group; △P < 0.05 compared to the saline group.

Osteoclasts are multinucleated giant cells that play a crucial role in bone resorption processes. TRAP staining was employed to evaluate bone resorption activity, osteoclast number, and distribution. TRAP-stained dental tissue sections (Fig 11a to d) displayed osteoclasts in the periodontal tissues of each group. Quantitative analysis of osteoclasts (Fig 11e) revealed that the control group exhibited the lowest number of osteoclasts in dental sections. Compared to the control group, the model group showed a significant increase in osteoclast numbers (P < 0.05). The saline group demonstrated a slight, non-significant reduction in osteoclast numbers compared to the model group (P > 0.05), while the TRP group showed a significant decrease in osteoclast numbers compared to the model group (P < 0.05), with a greater reduction than that observed in the saline group (P < 0.05).

### Effects of TRP on Inflammatory Cells in Rat Periodontal Tissues

**Fig 12a to h:**
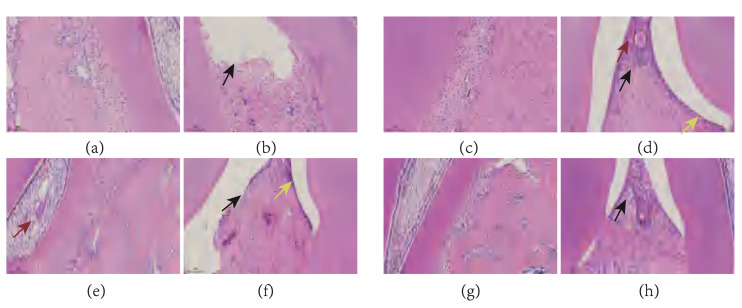
Representative histological images from each experimental group stained with hematoxylin and eosin (H&E). (a and b) control group showing normal odontoblasts, dense alveolar bone structure with normal osteocyte morphology, and focal gingival epithelial loss (black arrows). (c and d) model group (ligature + LPS) displaying regular odontoblast arrangement without abnormalities, uneven gingival epithelial thickness with irregular epithelial cell arrangement, focal epithelial cell necrosis with nuclear pyknosis or fragmentation (yellow arrows), mild inflammatory cell infiltration (black arrows), and minor vascular congestion and dilation (red arrows). (e and f) Saline group (ligature + LPS + saline irrigation) showing minor vascular dilation (red arrows), dense alveolar bone structure with normal osteocyte morphology, uneven gingival epithelial thickness with irregular epithelial cell arrangement, focal epithelial cell necrosis with nuclear pyknosis or fragmentation (yellow arrows), and focal epithelial loss (black arrows). (g and h) TRP group (ligature+LPS+TRP irrigation) demonstrating normal odontoblasts, dense alveolar bone structure, uneven gingival epithelial thickness with irregular epithelial cell arrangement, and mild inflammatory cell infiltration (black arrows). Images are representative of n = 6 animals per group.

Figure 12 shows representative histological images from each group. Tissue structures were clearly visible, with teeth composed of enamel, dentin, and cementum. The central pulp cavity contained blood vessels, connective tissue, and regularly arranged odontoblasts, with no apparent abnormalities observed. The alveolar bone structure appeared dense with normal morphology of osteocytes. The periodontal ligament, consisting of dense connective tissue, was visible between the teeth and alveolar bone.

The model group exhibited marked irregularity in gingival epithelial thickness with disorganised epithelial cell arrangement. Compared to the control group, focal epithelial cell necrosis was observed, accompanied by mild inflammatory cell infiltration and minor vascular congestion and dilation. The saline group showed focal epithelial cell necrosis with nuclear pyknosis or fragmentation, and focal epithelial loss. The TRP group, in addition to irregular epithelial cell arrangement, displayed uneven gingival epithelial thickness with irregular epithelial cell arrangement and mild inflammatory cell infiltration; however, vascular dilation showed improvement compared to the model and saline groups.

## DISCUSSION

In this study, we demonstrated that TRP exerts significant anti-inflammatory and antioxidative effects on periodontitis through inhibition of the *PI3K*/*AKT* signalling pathway, as revealed by integrated network pharmacology and experimental validation. Network analysis identified 129 potential therapeutic targets with *PI3K*/*AKT* emerging as the central regulatory hub, which was subsequently confirmed through *in vitro* and *in vivo* experiments. TRP treatment (20 μg/ml) effectively restored cell viability, reduced ROS levels, and downregulated key inflammatory mediators (IL-1β, IL-6, TNF-α, and MMP-8) in LPS-induced periodontal ligament fibroblasts. In the rat periodontitis model, TRP irrigation significantly preserved alveolar bone (BV/TV increased compared to the model group), reduced the RANKL/OPG ratio from 8.481 ± 1.56 to 2.3 ± 0.242, and decreased osteoclast numbers. These multi-level therapeutic effects position TRP as a promising natural agent for periodontal disease management.

The present study provides comprehensive evidence for the therapeutic potential of TRP in periodontitis through systematic integration of network pharmacology prediction with experimental validation. Our network pharmacology analysis identified 129 potential therapeutic targets of TRP intersecting with periodontitis-related genes, with the *PI3K*/*AKT* pathway emerging as a central hub in the regulatory network. This finding aligns with recent studies demonstrating that the *PI3K*/*AKT* pathway serves as a critical mediator of inflammatory responses in periodontal disease.^8^ The identification of hub genes, including *JUN*, *TP53*, *MYC*, *EGFR*, and *AKT1* was experimentally validated, showing significant downregulation following TRP treatment in LPS-stimulated cells. These findings are consistent with previous research showing that natural polyphenolic compounds can modulate multiple inflammatory signalling nodes simultaneously.^17^ The multi-target nature of TRP’s action, as revealed by our network analysis, provides a mechanistic explanation for its superior clinical efficacy compared to single-compound interventions reported in previous trials.^18^ Furthermore, our identification of oxidative stress response pathways among the top enriched biological processes corroborates earlier observations that TRP’s constituent plants are rich in antioxidant compounds, including gallic acid, ellagic acid, and chebulinic acid.^19^ The convergence of computational predictions with experimental validation strengthens the reliability of network pharmacology as a tool for elucidating complex herbal medicine mechanisms.

Our mechanistic investigations revealed that TRP exerts its anti-inflammatory effects primarily through suppression of the *PI3K/AKT* signalling cascade, as evidenced by decreased phosphorylation of *PI3K*, *AKT1*, and *AKT2*, coupled with upregulation of the negative regulator *PTEN*. The functional rescue experiments using *PI3K* activator (740Y-P) and inhibitor (LY294002) strongly suggest a causal relationship between *PI3K*/*AKT* inhibition and TRP’s anti-inflammatory effects. This mechanism is particularly relevant given recent evidence that hyperactivation of *PI3K*/*AKT* signalling in periodontal tissues contributes to sustained inflammation and impaired resolution of inflammatory responses.^13^ The observed reduction in inflammatory cytokines (IL-1β, IL-6, TNF-α) and MMP-8 following TRP treatment parallels findings from studies on other plant-derived anti-inflammatory compounds, suggesting shared mechanistic pathways.^10^ Notably, our study demonstrated that TRP’s antioxidative effects involve both direct ROS scavenging and upregulation of endogenous antioxidant defence systems, including SOD, CAT, and the Nrf2/HO-1 axis. This dual mechanism of action has been associated with superior therapeutic outcomes in inflammatory diseases compared to compounds targeting single pathways.^28^ The concentration-dependent effects observed (optimal at 20 μg/ml) provide crucial information for potential clinical translation, as therapeutic window determination is essential for herbal medicine standardisation.

The *in vivo* validation in our rat periodontitis model provided compelling evidence for TRP’s bone-protective effects, with significant preservation of alveolar bone volume and reduced osteoclast numbers compared to untreated controls. The modulation of the RANKL/OPG ratio, a critical determinant of bone homeostasis, suggests that TRP influences bone metabolism at the molecular level beyond its anti-inflammatory effects.^27^ The 72% reduction in RANKL/OPG ratio in the TRP group compared to the model group represents a substantial effect in this preclinical model, suggesting potential therapeutic relevance comparable in magnitude to effects reported with anti-resorptive agents in preclinical settings.^7^ Histological analysis revealed not only reduced inflammatory cell infiltration but also improved epithelial architecture and vascular normalisation, suggesting tissue-protective effects beyond simple anti-inflammatory action. These findings align with emerging concepts of resolution pharmacology, which emphasise the importance of active resolution processes rather than mere suppression of inflammation. The superior efficacy of TRP compared to saline irrigation alone highlights its potential as an adjunctive treatment to mechanical therapy. Importantly, the biocompatibility demonstrated in our cell viability assays, combined with the absence of adverse effects in the animal model, supports the safety profile of TRP reported in human trials.^23^ The translation of these findings to clinical practice could offer a cost-effective, accessible therapeutic option for periodontal disease management, particularly in resource-limited settings where conventional treatments may be unavailable.

Our mechanistic findings provide a scientific foundation for understanding the clinical efficacy of TRP mouthwash previously reported in human trials. Multiple randomised controlled trials have demonstrated that TRP mouthwash shows comparable efficacy to 0.2% chlorhexidine in reducing plaque accumulation and gingival inflammation in periodontitis patients.^18^ Bajaj and Tandon’s longitudinal study in school children further confirmed no significant difference between 0.6% TRP and 0.1% chlorhexidine mouthwash in controlling plaque, gingivitis, and microbial growth over a 9-month period.^5^ A systematic review and meta-analysis by AlJameel and Almalki, analysing seven randomised controlled trials, concluded that TRP mouthwash administration was significantly effective compared to chlorhexidine in treating plaque-induced gingivitis.^3^ Importantly, Pradeep and colleagues reported that TRP mouthwash significantly reduced plaque index, gingival index, and microbiologic colony counts at 7, 30, and 60 days without the adverse effects associated with chlorhexidine, such as tooth staining and taste alteration.^24^ These clinical observations align closely with our experimental findings demonstrating *PI3K*/*AKT* pathway inhibition, ROS reduction, and decreased inflammatory cytokine production by TRP. Our multi-level mechanistic data thus explain the molecular basis for TRP’s clinical anti-plaque and anti-inflammatory effects, while also clarifying why it achieves therapeutic benefits comparable to chlorhexidine through different underlying mechanisms – specifically through *PI3K*/*AKT* pathway modulation and antioxidative action rather than simple antimicrobial activity. This mechanistic understanding supports the potential for TRP to serve as a safer long-term alternative to chlorhexidine, addressing the critical need for effective adjunctive agents without the associated side effects.

Despite the comprehensive approach employed in this study, several limitations should be acknowledged. First, our *in vitro* experiments utilised only immortalised human periodontal ligament fibroblasts (hPDLFs), which may not fully recapitulate the complex cellular heterogeneity and interactions present in periodontal tissues, including gingival fibroblasts, epithelial cells, and immune cells. Importantly, periodontitis is a multifactorial disease involving critical interactions between resident tissue cells and infiltrating immune cells such as macrophages, neutrophils, and lymphocytes, which were not examined in our simplified cell culture model. Future studies incorporating co-culture systems with multiple cell types, particularly immune cells, would provide a more comprehensive understanding of TRP’s effects on the complex cellular crosstalk that characterises periodontal inflammation. The use of primary cells from periodontitis patients and healthy controls would provide more clinically relevant insights into TRP’s effects across different cellular phenotypes. Second, while our animal model successfully induced periodontitis through ligature placement and LPS injection, this acute inflammatory model differs from the chronic, polymicrobial nature of human periodontitis that develops over years. Specifically, our ligature-induced model with exogenous LPS application creates a relatively uniform and controlled inflammatory response, whereas human periodontitis involves dynamic shifts in complex polymicrobial biofilm communities (including *Porphyromonas gingivalis*, *Tannerella forsythia*, *Treponema denticola*, and numerous other species) that interact with host tissues over prolonged periods. This difference means our model may not fully replicate the chronic cycles of tissue destruction and attempted repair, nor the adaptive immune responses that characterise long-standing human periodontal disease. The 2-month treatment duration, though sufficient to observe bone changes, may not capture long-term effects or potential development of resistance to treatment. Third, the standardisation and batch-to-batch variability of TRP extract remains a challenge inherent to herbal medicine research, as the bioactive compound concentrations may vary depending on source, harvest conditions, and extraction methods. Although we used a standardised commercial preparation, comprehensive chemical profiling using LC-MS/MS or HPLC fingerprinting would strengthen the reproducibility of our findings. A critical limitation of our study is the absence of detailed chemical characterisation of the TRP extract used, including quantification of major bioactive compounds such as gallic acid, ellagic acid, chebulinic acid, and other polyphenols known to be present in *Terminalia chebula*, *Terminalia bellirica*, and *Phyllanthus emblica*. Without HPLC fingerprinting or LC-MS/MS analysis to establish the specific phytochemical profile and concentrations of active constituents in our extract, it is difficult to ensure exact reproducibility across different batches or compare our results with other studies usingdifferent TRP preparations. Future investigations should include comprehensive chemical characterisation with quantification of marker compounds to establish quality control standards and enable more precise correlation between specific phytochemical constituents and observed biological activities.

Fourth, while we focused on the *PI3K*/*AKT* pathway based on network pharmacology predictions, high-throughput approaches such as RNA sequencing or proteomics might reveal additional mechanisms and off-target effects not captured in our targeted approach. Finally, the translation of our findings from rat models to human clinical application requires careful consideration of species differences in periodontal anatomy, immune responses, and drug metabolism, necessitating future clinical trials to validate therapeutic efficacy and optimal dosing regimens in human subjects.

## CONCLUSION

In conclusion, this study successfully elucidated the molecular mechanisms underlying TRP’s therapeutic effects on periodontitis through an integrated approach combining network pharmacology prediction with comprehensive experimental validation. We demonstrated that TRP exerts potent anti-inflammatory and antioxidative effects primarily through inhibition of the *PI3K*/*AKT* signalling pathway, resulting in reduced inflammatory cytokine production, decreased oxidative stress, and preservation of alveolar bone integrity. The multi-target nature of TRP’s action, affecting 129 disease-related targets with *PI3K*/*AKT* as the central hub, provides a mechanistic basis for its clinical efficacy reported in previous trials. Our findings establish TRP as a promising natural therapeutic agent that could serve as an effective adjunctive treatment for periodontitis, offering particular value as a safe, accessible, and cost-effective option for long-term disease management. These results provide a scientific foundation for the development of TRP-based interventions in periodontal therapy and support further clinical trials to optimise dosing regimens and treatment protocols for human application.

### Acknowledgements

#### Declarations

##### Ethics approval and consent to participate

This study was conducted in accordance with ethical guidelines and approved protocols. The experimental animals were provided by the animal laboratory of Hangzhou Hunter Biotechnology, Inc. After obtaining the informed consent from the company to use these animals for research, the relevant experiments were conducted at the animal laboratory of Hangzhou Hunter Biotechnology, located in Hangzhou City, Zhejiang Province, China. Hunter Biotechnology, Inc. holds accreditation from the Association for Assessment and Accreditation of Laboratory Animal Care (AAALAC) International, the China National Accreditation Service for Conformity Assessment (CNAS), and the China Inspection Body and Laboratory Mandatory Approval (CMA). The Institutional Animal Care and Use Committee (IACUC) of Hunter Biotechnology, Inc. reviewed and approved the animal experiments (approval number: AAALAC 001458; approval date: 29 June 2012).

##### Availability of data and materials

The data used and analysed during the current study are available from the corresponding author upon reasonable request.
